# An experimental field study of inbreeding depression in an outcrossing invasive plant

**DOI:** 10.3389/fpls.2024.1393294

**Published:** 2024-08-29

**Authors:** Christopher M. Balogh, Spencer C. H. Barrett

**Affiliations:** Department of Ecology and Evolutionary Biology, University of Toronto, Toronto, ON, Canada

**Keywords:** colonization, competition, field experiment, inbreeding depression, invasive species, *Lythrum salicaria*

## Abstract

Inbreeding depression is likely to play an important role during biological invasion. But relatively few studies have investigated the fitness of selfed and outcrossed offspring in self-incompatible invasive plants in natural environments in their introduced range. Moreover, the majority of studies on inbreeding depression have investigated self-compatible species with mixed mating, and less is known about the intensity of inbreeding depression in outcrossing self-incompatible species. Here, we address these questions experimentally by comparing selfed and outcrossed progeny of purple loosestrife (*Lythrum salicaria*) over four growing seasons, including three under field conditions in a freshwater marsh in southern Ontario, Canada, a region where *L. salicaria* is highly invasive. The tristylous mating system of *L. salicaria* involves disassortative mating among floral morphs enforced by trimorphic incompatibility. However, owing to partial incompatibility, self-fertilized seed can be obtained by manual self-pollination thus facilitating comparisons of selfed and outcrossed progeny. We compared progeny with and without intraspecific competition from selfed or outcrossed neighbours and examined the influence of breeding treatment and competition on fitness correlates by measuring a range of life-history traits including: proportion of seeds germinating, days to germination, survival, proportion of plants flowering, time to flowering, vegetative mass, and inflorescence number and mass. We analysed data for each trait using functions from time series estimates of growth and two multiplicative estimates of fitness. We detected varying intensities of inbreeding depression for several traits in three of the four years of the experiment, including inflorescence mass and reproductive output. Cumulative inbreeding depression over four years averaged *δ* = 0.48 and 0.68, depending on the method used to estimate multiplicative fitness. The competition treatments did not significantly affect plant performance and the magnitude of inbreeding depression. Given the primarily outcrossing mating system of *L. salicaria* populations, the detection of inbreeding depression for several key life-history traits was as predicted by theory. Our results suggests that biparental inbreeding and low selfing in colonizing populations may have significant effects on demographic parameters such as population growth.

## Introduction

The amount of inbreeding in a population is a key determinant of fitness influencing its demography and growth rate. When the population structure of a species includes small isolated patches, individuals often mate with related individuals (biparental inbreeding), or if self-compatible they may reproduce via self-fertilization (reviewed in Lloyd, 1980; Barrett, 2011; Pannell, 2015). These forms of inbreeding increase homozygosity leading to the expression of deleterious recessive alleles normally sheltered in the heterozygous state in outcrossing populations, and this process causes inbreeding depression (*δ*) and a loss of fitness (Lande and Schemske, 1985; Charlesworth and Charlesworth, 1987; Barrett and Kohn, 1991; Charlesworth and Willis, 2009). Populations that are small and isolated are ubiquitous features of most colonizing species and individuals on the edge of an invasion front may undergo serial bottlenecks causing the loss of heterozygosity and reduced genetic variation (Shigesada and Kawasaki, 1997; Eckert et al., 2008; Excoffier et al., 2009). Thus, the extent to which inbreeding influences the fitness of populations in colonizing species is a key question in invasion biology and for contemporary evolution.

Inbreeding depression is not a static property of individuals or populations but rather may vary in intensity depending on the history of inbreeding in populations (Lande and Schemske, 1985; Barrett and Charlesworth, 1991; Carr and Dudash, 1997; Leimu et al., 2008), ecological context and the life-history traits investigated (Dudash, 1990; Husband and Schemske, 1996; Angeloni et al., 2011; Cheptou and Donoghue, 2011; Murren and Dudash, 2012). It has often been suggested that adverse and/or stressful environmental conditions should reduce the performance of inbred offspring more strongly than outbred offspring, thus increasing the strength of inbreeding depression and there is empirical support for this hypothesis (Cheptou et al., 2000; Kariyat et al., 2011; Sander and Matthies, 2016; Sandner et al., 2021). Although a meta-analysis of numerous plant and animal taxa reported that stress conditions increased inbreeding depression by 69% overall (Armbruster and Reed, 2005), this response was not universal across all studies.

A key issue in evaluating the influence of adverse conditions on the intensity of inbreeding depression is what form of stress is being evaluated and whether this involves abiotic or biotic challenges (Leimu et al., 2008). For example, variation in the importance of density-dependent biotic interactions has been shown to influence the strength of inbreeding depression (Yun and Agrawal, 2014). In plants, such effects are sometimes expressed as dominance and suppression under competitive conditions in which outcrossed progeny pre-empt resources from selfed progeny reducing the growth of selfed progeny and causing elevated inbreeding depression (Schmitt et al., 1987; Schmitt and Ehrhardt, 1990). Because of the immobility of plants, the influence of inbreeding on fitness is likely to be particularly sensitive to the density and breeding history of neighbours.

Biological invasions can act as natural experiments allowing the investigation of evolutionary processes during contemporary time. Invasive species frequently encounter novel environments during range expansion leading to natural selection and local adaptation (Maron et al., 2004; Colautti and Barrett, 2013; Odour et al., 2016). However, serial bottlenecks and founder events are also an intrinsic feature of the invasion process and are often accompanied by increased inbreeding and the loss of genetic diversity (Barton and Charlesworth, 1984; Dlugosch and Parker, 2008; Peischl and Excoffier, 2015, but see Estoup et al., 2016). Despite the diverse processes that can potentially influence the fitness of colonizing populations there have been relatively few attempts to quantify inbreeding depression in invasive species (but see Parisod et al., 2005; Facon et al., 2011; Mullarkey et al., 2013; Rosche et al., 2017; Schreiber et al., 2019a), and fewer have investigated how inbreeding depression may vary with environmental conditions and biotic challenges (but see Garcia-Serrano et al., 2008; Schrieber et al., 2019b), despite the possibility that this might influence evolution during biological invasion (Schrieber and Lachmuth, 2017).

*Lythrum salicaria* (Lythraceae) is an autotetraploid, outcrossing, herbaceous, perennial native to wetlands, ditches and river edges in Eurasia. Recently, the species has become highly invasive, especially in eastern North America where it has spread rapidly over the past 150 years since its introduction to the eastern seaboard of the U.S.A (Thompson et al., 1987). Invasive populations vary considerably in size and density, from small isolated populations to very large monospecific standards comprised of many thousands of plants, often at high density (Eckert and Barrett, 1992; Balogh and Barrett, 2016). Plants do not reproduce by clonal growth and thus all regeneration in populations is by seed (Yakimowski et al., 2005). The maximum age of plants has not been established by demographic studies, but individuals often persist for at least 15 or more years (Thompson et al., 1987; S. C. H. Barrett *pers. observ*.). Flowers of *L. salicaria* are predominantly bee-pollinated, particularly by *Apis mellifera* and *Bombus* spp., although other pollinators such as butterflies, wasps, and occasional hummingbirds visit flowers for pollen/and or nectar (Thompson et al., 1987; King and Sargent, 2012). Local adaptation in flowering time and plant stature has been demonstrated in both the native and invasive range of *L. salicaria* based on the length of the growing season (Olsson and Ågren, 2002; Bastlová et al., 2004; Colautti and Barrett, 2013). Because *Lythrum salicaria* is still undergoing invasion to new areas in North America (e.g. especially in Ontario, Canada), the species is a desirable study system for examining the extent of inbreeding depression in colonizing populations.

The contemporary evolution of local adaptation in invasive populations of *L. salicaria* in eastern North America has undoubtedly been facilitated by considerable amounts of quantitative genetic variation for key life-history traits (Colautti and Barrett, 2011). The maintenance of this variation is promoted by the largely outcrossed mating system of populations. *Lythrum salicaria* is tristylous and possesses a trimorphic incompatibility system; progeny tests of open-pollinated families have confirmed that this form of incompatibility enforces high rates of disassortative (intermorph) mating in populations (Balogh and Barrett, 2018a). However, in most populations of *L. salicaria* partially self-incompatible individuals capable of limited seed set by selfing have been detected following controlled hand-pollination (Balogh and Barrett, 2018b) or through the seed set of isolated plants (Stout, 1923). It is unclear to what extent the occurrence of standing genetic variation for partial self-incompatibility in *L. salicaria* influences mating patterns in large populations; however, in small isolated colonizing populations where reproductive assurance may be important, selfing and intramorph mating seem likely to occur (Balogh and Barrett, 2018a). Regardless of the potential adaptive significance of partial self-incompatibility in colonizing populations, the occurrence of some degree of self-compatibility in *L. salicaria* facilitates the generation of selfed offspring and we exploited this feature of the species to enable us to measure inbreeding depression.

Here, we investigate the expression of inbreeding depression in *L. salicaria* for a range of life-history traits by comparing the performance of selfed and outcrossed families under glasshouse and field conditions. The only previous study of inbreeding depression in *L. salicaria* investigated germination and seedling traits over a five-week period and reported values of *δ* ranging from 0.44-0.64 (O’Neil, 1994). Our study extended the time period in which inbreeding depression was measured and addressed the following specific questions. 1) How strong is inbreeding depression in *L. salicaria* and what is the cumulative expression of inbreeding depression over four growing seasons? We predicted that owing to the primarily outcrossed mating system of *L. salicaria*, inbreeding depression should be evident, although frequent colonizing episodes, bottlenecks and biparental inbreeding might potentially reduce genetic load and hence the overall strength of inbreeding depression (Lande and Schemske, 1985; Pujol et al., 2009). 2) Does the presence of intraspecific selfed and outcrossed competitors influence the magnitude of inbreeding depression in life-history traits? We addressed this question by adding either a selfed or an outcrossed competitor to selected pots in our experiment and determining their influence on a focal selfed or outcrossed plants in the same pot. There were two predictions from the competition treatments: i) outcrossed focal plants should experience a greater reduction in performance when competing against outcrossed than selfed offspring; ii). selfed focal plants should experience a greater reduction in performance when competing against outcrossed than selfed offspring. These predictions would be detected as an interaction between competitive environment and breeding treatment in our analysis. 3) When during the season does inbreeding depression most strongly influence the growth rate of individuals? We evaluated this by examining changes in plant height during the growing season (June – September), fitting the data to several nonlinear growth-rate functions each year, and comparing the average growth rates between treatments in each year.

## Materials and methods

### Source material

*Lythrum salicaria* possesses a trimorphic incompatibility system with variable expression enabling some plants, especially of the mid-styled morph, to produce self-fertilized seed following hand self-pollination (Stout, 1923; Balogh and Barrett, 2018b). We obtained selfed and outcrossed families of *L. salicaria* from 29 maternal plants that set more than 10 seeds following self-pollination in the study by Balogh and Barrett (2018b). The plants originated from four large populations in the Greater Toronto Area, Ontario, Canada (HUM at latitude and longitude 43.6227, -79.473903, CDV at 43.689736, -79.42395, RRV at 43.813778, -79.488703, and DON at 43.78075, -79.3699), all of which were trimorphic and contained ~1000 individuals. The offspring used in the experiment were obtained from self- and cross-pollinations of 5 L-, 17 M- and 7 S-morph maternal parents and all parents produced both selfed and outcrossed progeny. We considered the parental individuals used in the crossing program as belonging to a single sample, given the close proximity of populations to one another in the Toronto area, the likelihood of gene flow between them, and their relatively recent origin (past 30 years). Thus, we did not investigate population-level variation in inbreeding depression in our study.

### Comparison of fitness components under glasshouse conditions

In early April 2014, we filled five 200-cell germination trays with Promix-BX potting mix and planted 10 self- and 10 cross-fertilized seeds per family blocked across the trays. The trays were placed under vented plastic covers on a bench in a glasshouse at the Earth Sciences Centre, University of Toronto and maintained between 15-25°C until germination. We recorded whether each seed germinated, the time of germination, and the survival of each seedling after germination.

**Figure 1:**
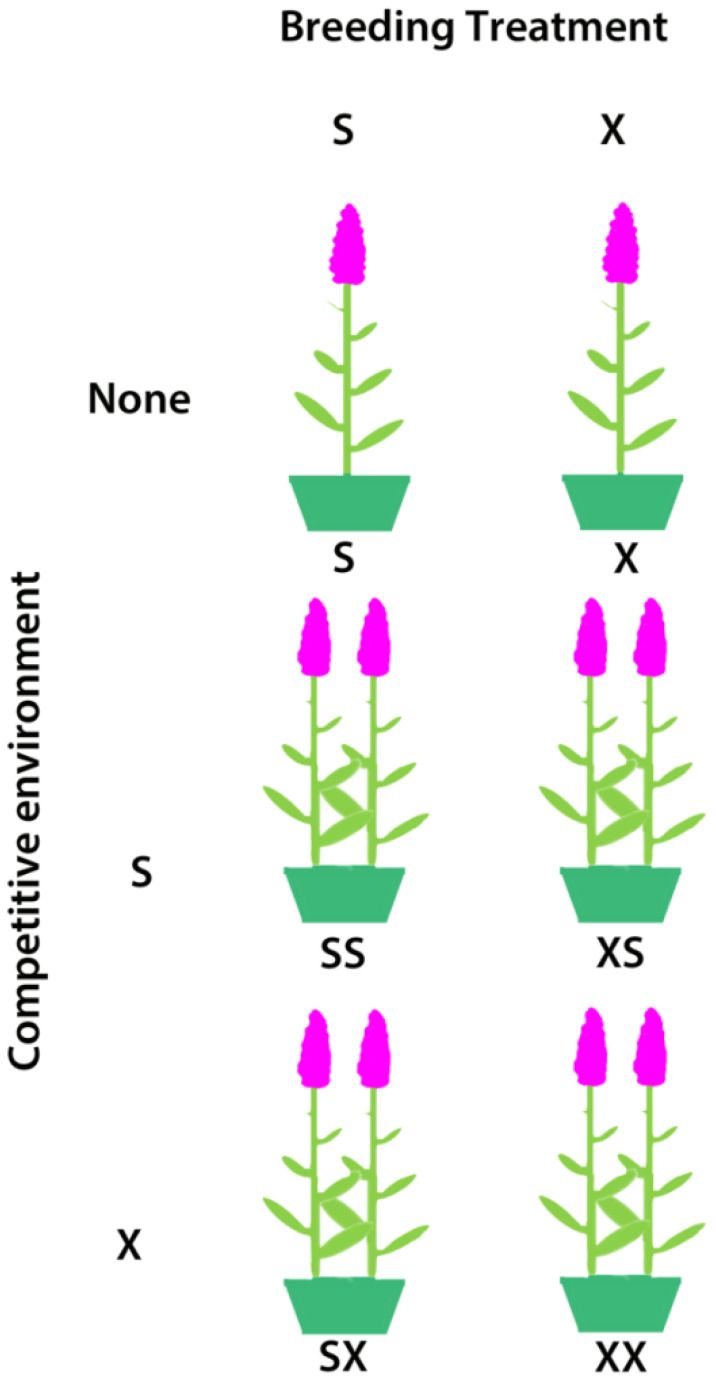
The competition and breeding treatments used in the inbreeding depression experiment on *Lythum salicaria*. In each pot is a focal individual with and without a S (selfed) or X (outcrossed) competitor. Focal plants are always on the left in this illustration but were arranged haphazardly in the experiment.

On May 8-11, after seedlings had developed true leaves and around the time when *L. salicaria* begins to grow in southern Ontario, we assigned three self- and three cross-fertilized progeny (hereafter S and X, respectively) from the 29 families which produced at least six offspring per breeding treatment to serve as focal plants in all stages of the inbreeding depression experiment. We assigned each of the selected seedlings to one of three ‘competition environments’ by transplanting individuals: 1) singly in a pot; 2) in a pot with a selfed competitor; 3) in a pot with an outcrossed competitor. Thus, we produced a total of six treatments (S, X, XS, XX, SX, SS) where the first letter represents the breeding treatment of the focal plant and the second letter (or lack thereof) represents the breeding treatment (or absence) of the competitor (**Figure 1**). The non-focal plant in pots with two plants originated from a different family than the focal plant.

**Figure 2:**
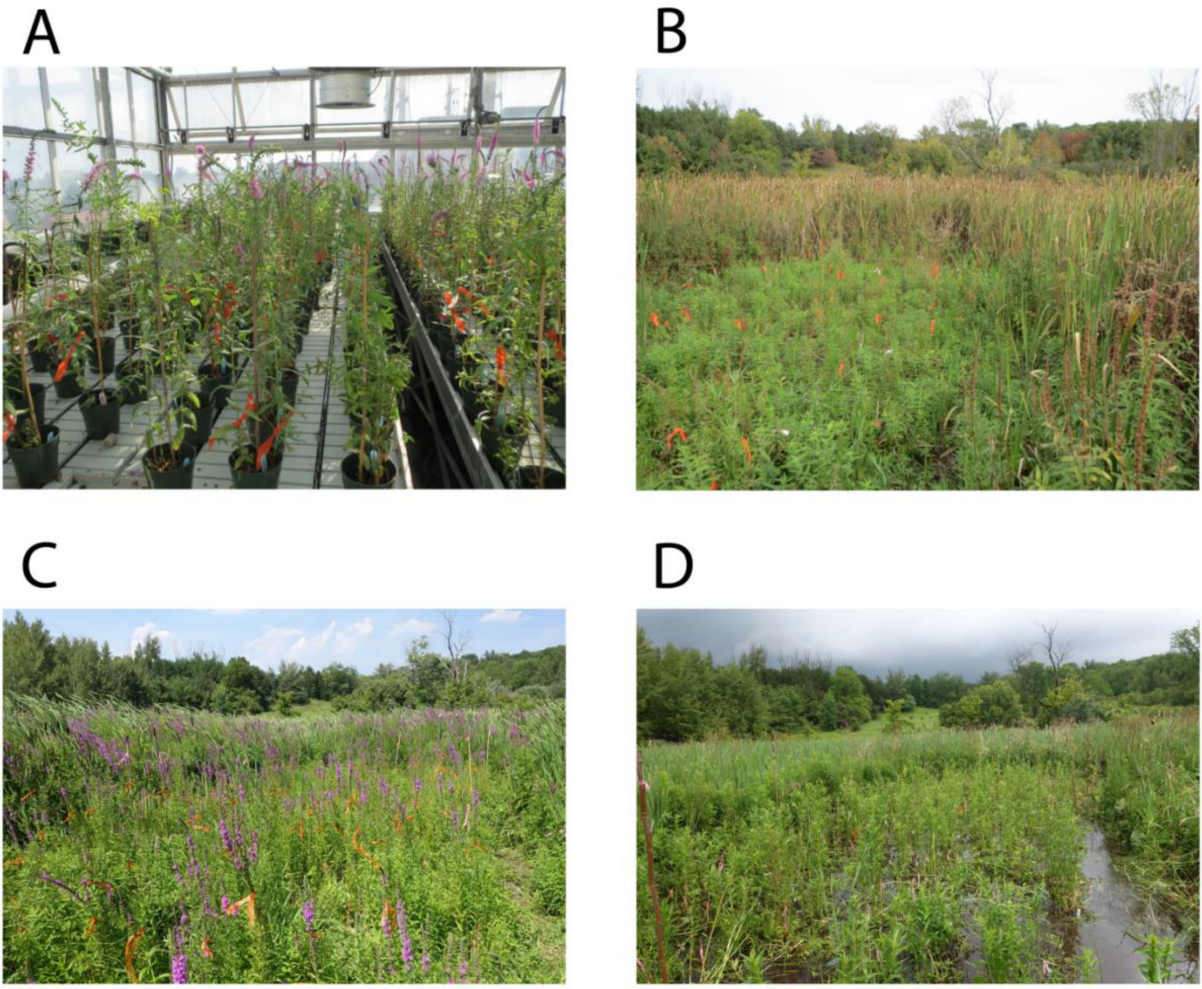
Images of the inbreeding depression experiment on *Lythrum salicaria* over four growing seasons (2014-7). **(A)** Earth Sciences glasshouse and **(B-D)** Koffler Scientific Reserve. **(A)** July 2014, flowering; **(B)** Early October 2015, post flowering; **(C)** Early August 2016, flowering; **(D)** Late June 2017, pre-flowering.

We used 12.5 cm (5” standard) pots with Promix-BX media and randomly blocked plants by family into six blocks on two flooded (~2 cm depth) benches in the glasshouse (**Figure 2A**). We maintained water in the benches at all times and added water-soluble fertilizer (14N-14P-14K) every two weeks following the manufacturer’s instructions. Throughout the summer, we measured plant height from the soil surface approximately every two weeks and recorded the flowering date of each plant as days since transplanting was completed (May 11). When plants ceased flowering in early October, corresponding to the end of the growing season, we recorded each plant’s survival since transplant, flowering, height of each individual measured from the soil surface to the tallest point, length of the longest inflorescence on each individual, the basal stem diameter from two positions at 90-degree angles from each other (with the two measures averaged), the number of vegetative stems at the base of the plant, and the number of inflorescences on each plant. We then harvested the above ground tissue from each plant, dried it in a 50°C degree drying oven for 30 days, and measured the dry vegetative and total inflorescence mass of each plant.

### Comparison of fitness components under field conditions

In spring 2015, we transported all pots in the glasshouse experiment to the Koffler Scientific Reserve (44.02610 N, 79.53549 W) in Newmarket, Ontario, Canada where they were placed into a common garden field experiment for three flowering seasons (2015-7; **Figures 2B–D**). This common garden is approximately 30 km north of the Toronto populations from which the parental plants were sampled. Pots were randomized and sunk into saturated soil in a disturbed freshwater marsh dominated by *Typha latifolia* and a few wild *L. salicaria* plants. The experimental plot measured 4.5 by 10 m and was cleared of above ground vegetation at planting and at the beginning of each growing season, and two subsequent times in July and September in each growing season, to facilitate the location of marked treatment plants. We visited the plot twice per week during the growing season and the day of first flowering for all plants was estimated. In 2015 and 2017 the site was flooded owing to natural rainfall and watering was not required; however, in 2016 drought conditions prevailed and we added approximately 30-40 gallons of water to the plants and soil around them on a weekly basis.

On four occasions during the growing seasons of 2015 and 2016 and on three occasions in 2017, we measured plant height from soil surface to the tallest point, the number of vegetative stems at the base, and the length of the longest inflorescence. In early October of each year when plants ceased growth, but had not yet dropped leaves, we harvested the above-ground biomass (inflorescence and vegetative tissue), dried, and weighed these tissues, as described in the preceding section. We also recorded whether or not each plant survived or flowered in each of the years. After the completion of the experiment in October 2017, we removed all pots from the field and destroyed all plants. The sample size at the end of each year varied due to plant death in subsequent seasons (*n* = 176, 174, 121 in 2015, 2016, 2017, respectively).

### Analysis of germination and end-of-season life-history trait data

We used the end-of-season trait data from the glasshouse and field as approximations of individual fitness components and used values in each year to determine multiplicative fitness correlates for each treatment. We performed all statistical analyses in R version 3.3.2 ‘Sincere Pumpkin Patch’ (R Core Team, 2016) and used linear mixed-effects models (lme4) where appropriate, and model checking was based on visual inspection of residuals versus fitted plots. We estimated the overall correlations between each trait measured at the end of each year (2014-17), measured the correlations between traits in each of the six breeding and competitive treatment combinations, and visualized the correlations between these values in the package ‘corrplot’ (Wei and Simko, 2017) (**Supplementary Figure 1**). Significant correlations occurred between traits overall; however, the correlations differed significantly between the six treatments. As a result, we were not able to select a single trait correlate of plant biomass at the end of each year, nor was it possible to use one easily measured variable as a surrogate for another. Therefore, each year, we used plant survival, whether or not a plant flowered, date of flowering, and total inflorescence mass as the end-of-year fitness correlates. We used plant height as a proxy for plant vigour in the non-linear functions of growth (see below).

We analysed germination data using mixed models with binomial (for germination and survival) or Poisson (for days to germination) residual distributions with breeding treatment (selfed or outcrossed) as a fixed variable and family and germination tray as random variables. We analysed the end-of-year data for each of the four years following mixed-modelling protocols with family as a random variable and three structures for the fixed variables: breeding treatment, competitive environment, or these variables plus their interaction. We performed mixed models for untransformed inflorescence mass in 2014, log-transformed (to meet model assumptions) inflorescence mass in 2015 to 2017, and the log-transformed number of days from the 2014 final transplant date (May 11, see earlier) to flowering date in each year to maintain a standard for potential comparison between years. We applied a generalized linear mixed model with binomial variables for survival and flowering in each year.

We used the package estimated marginal means (‘emmeans’, Lenth, 2018) to obtain the mean and 95% confidence interval estimates of each trait in each set of treatments. We also used the R package ‘emmeans’ to calculate various relative performance (*RP*) metrics. We estimated the relative performance between inbred and outcrossed progeny, inbreeding depression (*δ*), as *δ* = 1 - *w_s_*_/_ *w_o_* (*w_s_* = selfed progeny performance and *w_o_* = outcrossed progeny performance) if *w_o_* > *w_s_* or *δ* = *w_s_*_/_ *w_o_ –* 1 if *w_s_* > *w_o_* (following Ågren and Schemske, 1993). We investigated whether there was a reduction in performance caused by competition environment with an analogous set of metrics: 1 - *w_scomp_/w_none_* if *w_none_* > *w_scomp_*, 1 - *w_ocomp_/w_none_* if *w_none_* > *w_ocomp_*, and 1 - *w_ocomp_*/*w_scomp_* if *w_scomp_* > *w_ocomp_* (where *w_none_*, *w_scomp,_* and *w_ocomp_* represent the performance of all focal plants without competitors, with self-fertilized competitors, and with cross-fertilized competitors, respectively) and negative values if performance is in the opposite direction: *w_none/_w_scomp_ –* 1 if *w_none_* < *w_scomp_*, *w_none/_w_ocomp_* – 1 if *w_none_* < *w_xcomp_*, and *w_scomp_/w_ocomp_* – 1 if *w_ocomp_* < *w_scomp_*. For both of these metrics, non-significant differences in performance are equal to 0 whereas significant differences are different from zero. Competitive treatments rarely caused a significant change in plant performance, but in the cases where competition had a significant effect, we calculated inbreeding depression independently for plants with no competitor, a selfed competitor, and an outcrossed competitor, and statistically compared the difference in these ratios using log-transformed data if the original data was continuous, following Johnston and Schoen (1994).

### Analysis of growth via non-linear time series

The time at which inbreeding depression affects growth rate can alter competitive performance between selfed and outcrossed progeny in a multiplicative fashion. In particular, the expression of inbreeding depression in early-life vigour may exacerbate later differences in plant fitness correlates via dominance and suppression, which occurs when outcrossed progeny pre-empt resources and increase the expression of inbreeding depression in selfed plants (Schmitt et al., 1987; Schmitt and Ehrhardt, 1990). We fitted all nonlinear growth curves using the R package ‘nlme’ (Pinheiro et al., 2018) and controlled for multiple measures on individuals by defining each plant as a ‘group’ random factor term, which directed the model to estimate fixed model terms based upon each individual plant’s growth curve. We standardized measurement dates as days from May 11 in each year, as discussed earlier. These controls allowed comparisons within years between treatments and between years for model behaviour.

We fitted the mean height of all plants (*y*) at each measurement time (*t*) in 2014-2016 to each of four asymptotic non-linear growth models presented by Paine et al. (2012) and compared the AIC of each model to select an optimal model. We did not collect data at enough individual time points during 2017 to produce a nonlinear model. These four models consist of different parameters; the ‘monomolecular model’ (Richards, 1959):


(1) $$y_{\left(t\right)}=Asymp+\left(R_{0}-Asymp\right)*exp^{-\exp^{lrc}*time}$$

In which *Asymp* represents *y* at high values of *t*, *R_0_* determines *y* when *t* is 0, and *lrc* determines the growth rate in the model; the ‘logistic model’ (Hunt, 1981; Zeide, 1993):


(2) $$y_{\left(t\right)}=\;\frac{Asymp}{1+exp^{\frac{xmid-time}{scal}}}$$

In which *Asymp* defines *y* at high values of *t*, *xmid* is the time *t* at which *y* value is ½ *Asymp*, and *scal* is a parameter relating the model *y* values to *t*; the ‘Gompertz model’ (Gompertz, 1825; Winsor, 1932):


(3) $$y_{\left(t\right)}=Asymp*exp^{-b2*b3^{\;time}\;}$$

In which *Asymp* is equal to *y* when *t* is large, *b2* is a parameter defining *y* when *t* = 0, and *b3* scales the model relative to *t* and the ‘four-part-logistic model’ (Hunt, 1981; Zeide, 1993):


(4) $$y_{\left(t\right)}=A+\frac{\left(B-A\right)}{1+exp^{\frac{xmid-time}{scale}}}$$

In which *A* determines *y* when *t* is small, *B* determines the maximum value of *y* when *t* is large, *xmid* is the value of *t* at which *y* is exactly halfway between *A* and *B*, and *scal* adjusts positioning of the model relative to *t*. We fit the periodic height measurements from each year to each of these four models and measured the AIC of each fit. We analysed each year using the growth curve that possessed the lowest AIC.

We produced mixed models fitting the growth curve with the lowest AIC for each year following three formats of fixed variables: breeding treatment by growth curve parameters, competitive environment by growth curve parameters, and these factors plus their interaction by parameters. If the models did not converge with breeding and competitive environment treatment attached to each model parameter, we removed the breeding and competitive environment covariate from those parameters. We measured the significance of the breeding and competition terms on model parameters using the ‘nlme’ package’s marginal values ANOVA. We also used the R package ‘emmeans’ to calculate the effects of each breeding and competitive treatment on performance based on the estimated model means of the nonlinear model parameters.

We analysed the intensity and timing of growth rate differences between models using the average growth rate (*AGR*) from model curves calculated independently in each of the breeding and competitive environmental treatments. We independently modelled the growth curves from each breeding treatment, competitive environment, and the six combinations of these variables in each of the years studied. We used the distribution of means, variances, and co-variances in parameters from each subset of the model to produce 1000 simulated growth curves from each distribution of parameter sets and then extracted the 95% confidence intervals of model terms. Using the first derivative of the growth curves and the 95% CI of model terms, we produced estimates of *AGR* across model time and its 95% confidence interval (centimetres of growth per day) from each breeding treatment, competitive environment, and the interaction of these terms continuously from *t* = 0 to *t* = 150 days and estimated the inbreeding depression in average growth rate across these time periods.

### Comparisons of multiplicative fitness function

Inbreeding depression depends on multiplicative interactions between life-history traits across the lifetime of an organism. In perennial organisms, year-to-year survival and reproductive output determine an organism’s lifetime reproductive fitness and may vary from year-to-year depending upon changes in environmental conditions (Johnston and Schoen, 1994; Husband and Schemske, 1996). We calculated two multiplicative fitness functions for selected trait data – one was based on family-level fitness components whereas for the other we simulated organisms and assigned each with fitness correlates by resampling from the observed data. We used two models because small family size prevented us from investigating the interactions between breeding treatment and competitive environment in the family-based measure, whereas a simulated distribution enabled us to investigate breeding and competitive effects, and their interaction. In the family-based model, we multiplied the mean germination percent expressed by each family in 2014 by the mean proportion of plants surviving, proportion of plants flowering, and the mean inflorescence mass for plants producing inflorescences from each of the years. We added these terms for each treatment in each family to produce a value of ‘cumulative reproductive success’. We then calculated an index of relative performance between selfed and outcrossed multiplicative fitness correlates within each family - if *w_o_* > *w_s_*, we calculated the inbreeding depression for those plants as 1 – *w_s_*/*w_o_*; however, if *w_s_* > *w_o_*, we calculated the relative performance index as *w_o_*/*w_s_* – 1, which generated a negative inbreeding depression value symmetrical to the positive inbreeding depression value. We measured the mean inbreeding depression and standard error using family mean inbreeding depression values.

Calculating the multiplicative fitness function via resampling required additional specialization in model production. To produce a protocol for individuals in the resampling simulation we needed to maintain a similar simulated population size (*n*) to the size of the observed populations. Therefore, the resampling fitness function in R simulated 246 self- and outcrossed-seeds which, on average, provided the same sample size of adult plants as in the study (*n* = ~176 in 2014). We started with these simulated seeds and sampled germination success, survival, flowering success, and inflorescence mass for each simulated plant from the distributions in the observed data for each treatment and for each year of data. If a simulated plant failed to flower in a year it received NA (not applicable) for inflorescence mass; if an individual failed to survive it neither flowered nor produced stems in subsequent years. We simulated 5000 runs using this protocol and calculated multiplicative fitness for each of the six treatments in each year as proportion germinated x proportion which survived x proportion flowering x mean inflorescence mass. We additionally produced a cumulative inflorescence mass for each plant over the four simulated years as the ‘cumulative output’ function. We then examined the influence of breeding treatment, competitive treatment, and these values plus their interaction on the expression of relative performance, as described for the end-of-season data.

## Results

### Comparisons under glasshouse conditions

**Figure 3:**
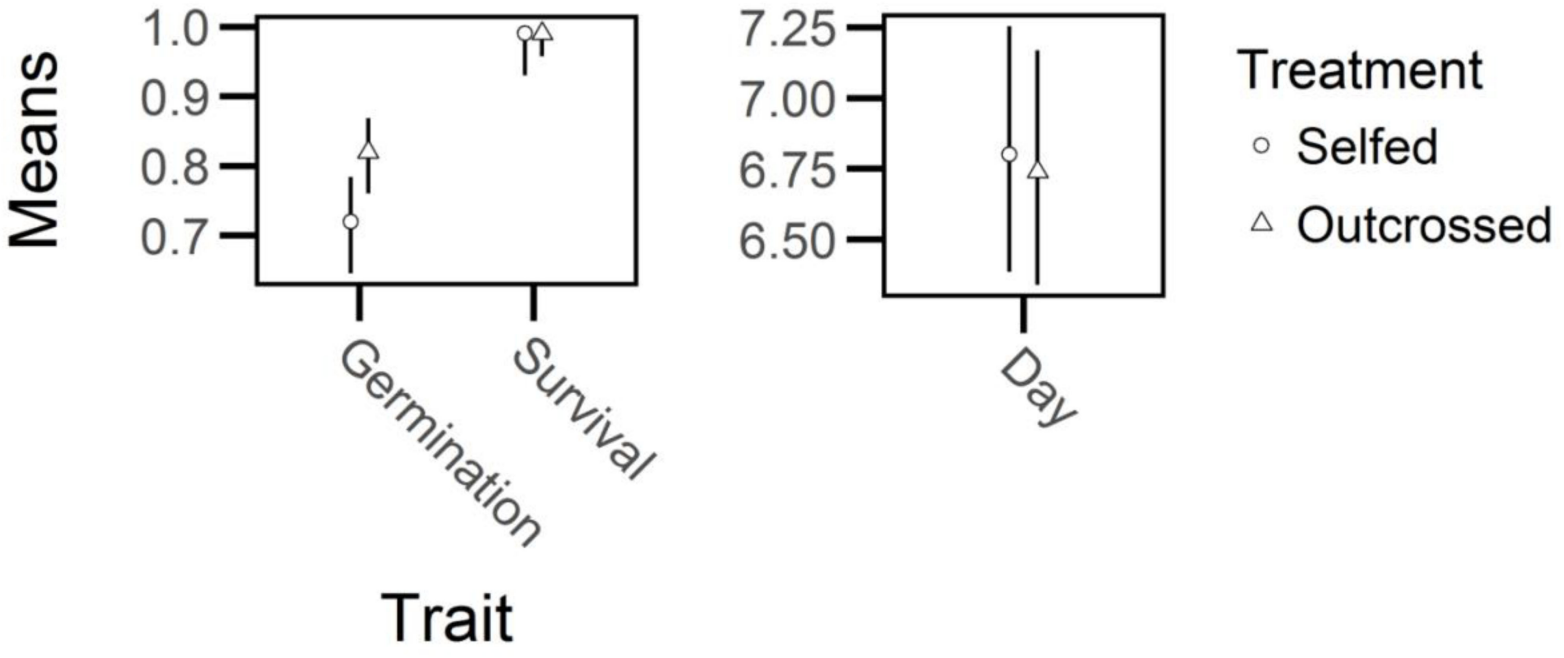
Marginal estimated means and 95% confidence intervals (bars) of trait values in early life of selfed- and outcrossed-individuals of *Lythrum salicaria*. Left: binomial traits (germination frequency and survival after germination); only germination frequency was significantly different between breeding treatments. Right: days to germination (‘Day’; note the difference in the y-axis scale relative to the left panel) was not significantly different between the treatments.

**Figure 4:**
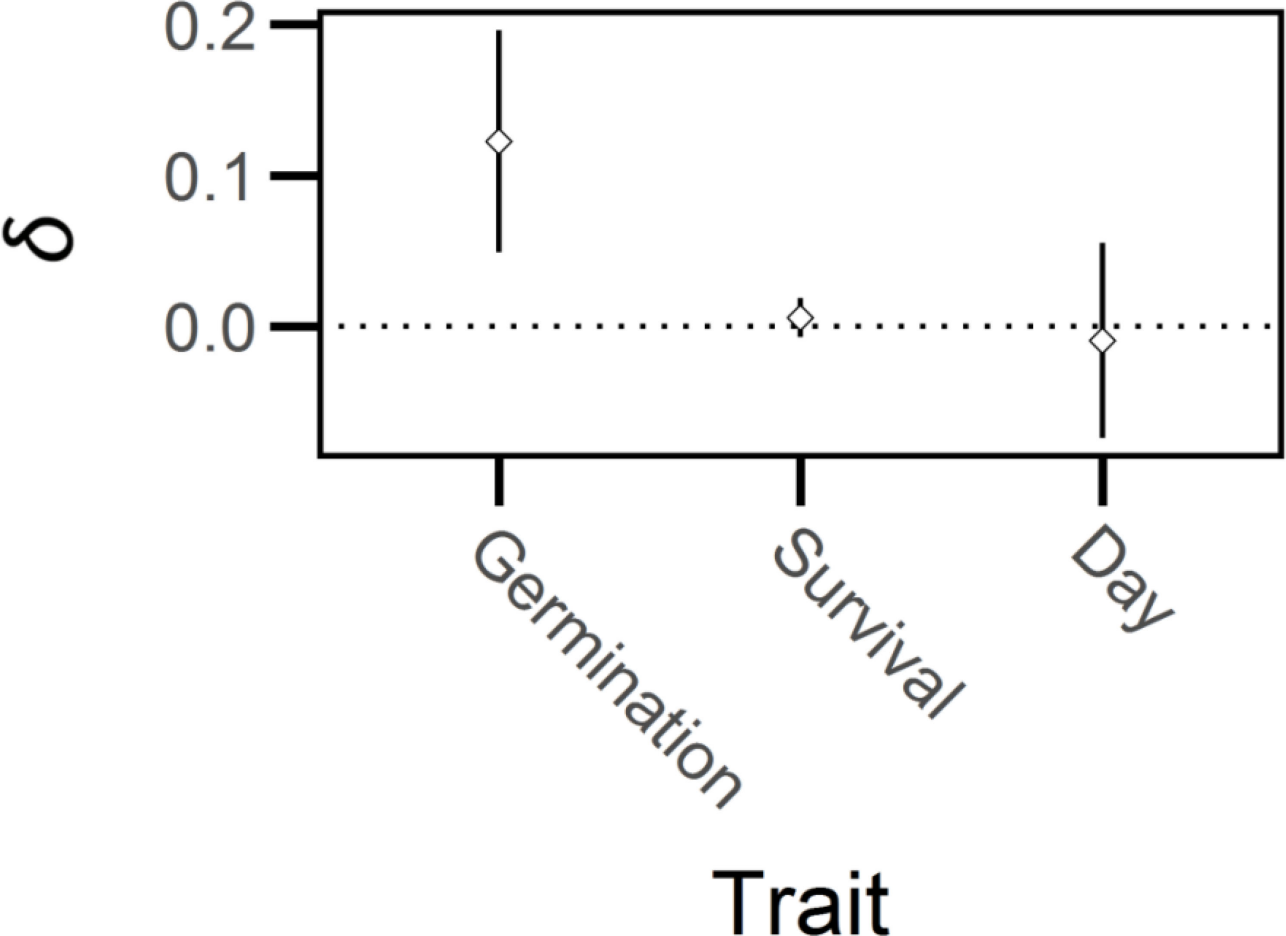
Mean inbreeding depression (*δ*) and 95% confidence intervals (bars) for early-life traits of *Lythrum salicaria.* Traits are germination frequency, survival after germination and days until germination, ‘Day’. There was weak but significant inbreeding depression for seed germination but not for survival after germination or days to germination.

Outcrossed seed exhibited a 10 percent increase in germination rate relative to self-fertilized seed (*X^2^* = 11, *df* = 1, *P* < 0.01; **Figure 3**) but there were no significant effects of breeding treatment on days to germination or on the survival of seedlings that germinated (days to germination: *X^2^* = 0.09, *df* = 1, *P* > 0.75; survival of seedlings: *X^2^* = 1.71, *df* = 1, *P* > 0.15). Thus, germination frequency experienced significant, but relatively weak, inbreeding depression (*δ* = 0.12, 95% CI = 0.05-0.20, **Figure 4**) whereas days to germination and survival after germination were not influenced by breeding treatment.

**Figure 5:**
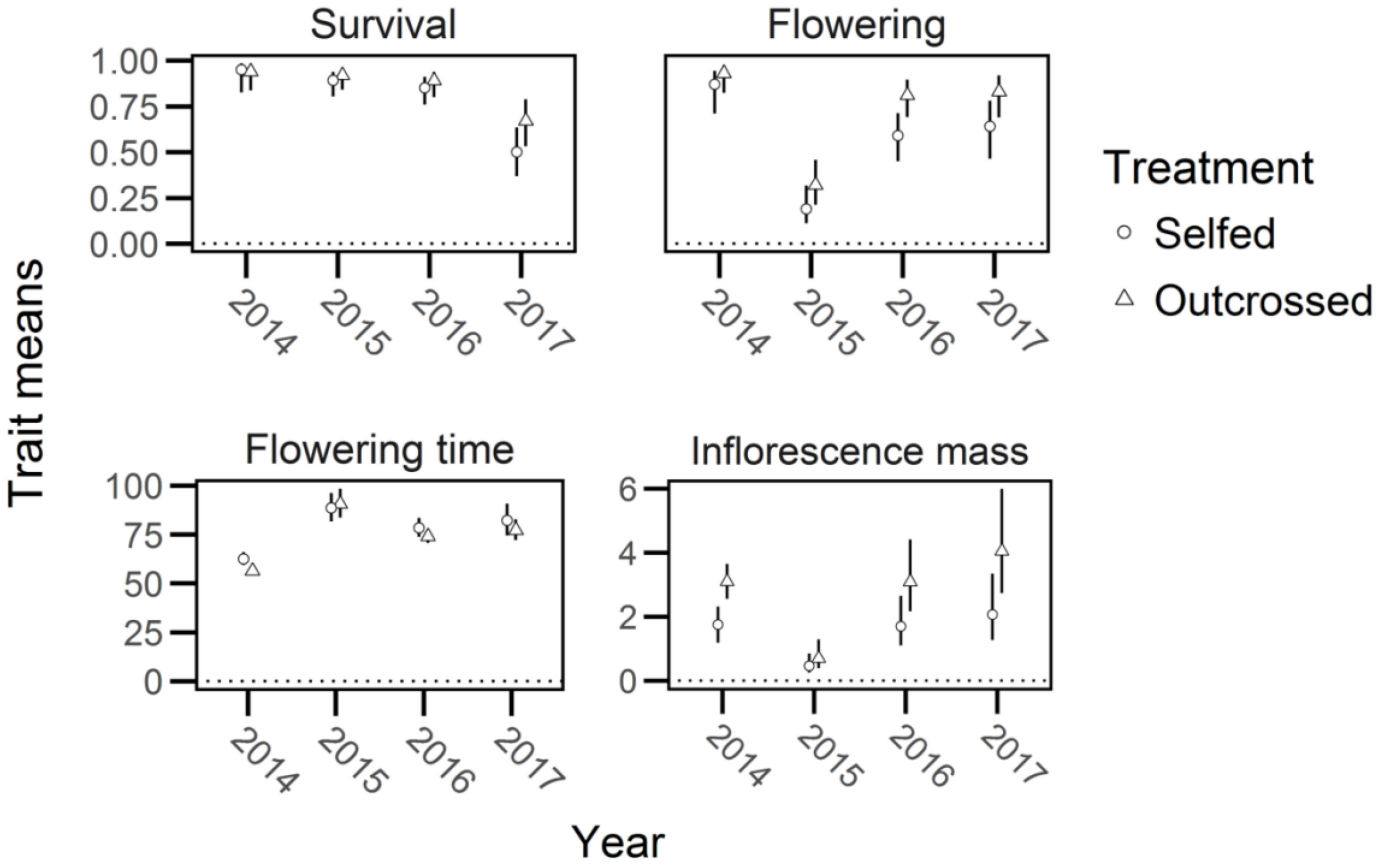
The mean and 95% confidence intervals (bars) for trait values of selfed and outcrossed progeny in *Lythrum salicaria* from 2014 to 2017. Values are depicted for survival, proportion of plants flowering (‘flowering’), flowering time, and inflorescence mass (note the differences in y-axis scales for flowering time and inflorescence mass). The largest difference in means was found in inflorescence mass for 2014, 2016, and 2017. Other mean values are relatively similar to each other and show no evidence of significant inbreeding depression.

**Figure 6:**
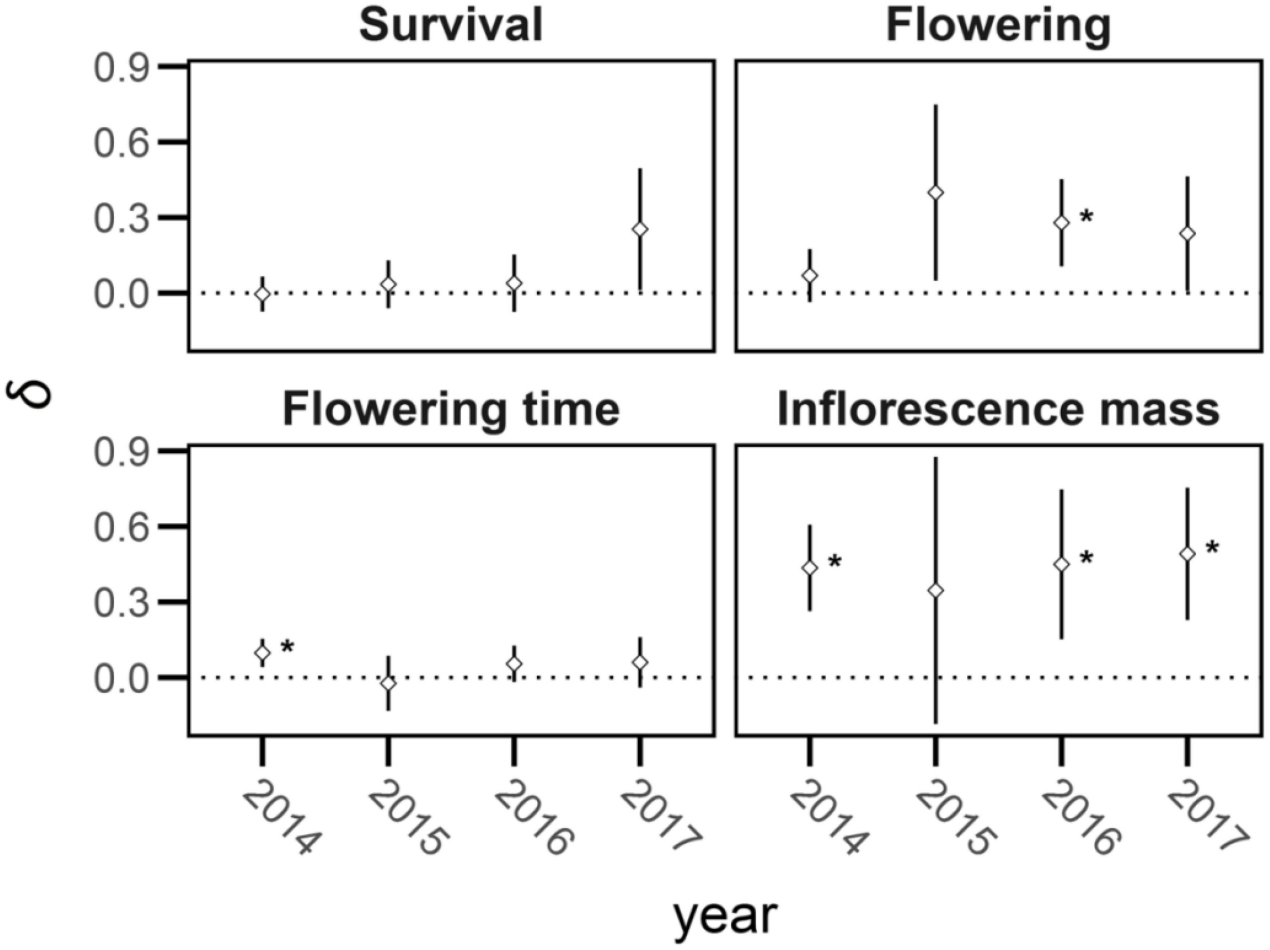
Mean inbreeding depression (*δ*) and 95% confidence intervals (bars) of life-history traits in *Lythrum salicaria*. Traits are survival, proportion flowering (‘flowering’), flowering time, and inflorescence mass in each year of the experiment (2014 – 2017). There was marginally significant inbreeding depression in survival in 2017 but not in other years and a significant difference in likelihood of flowering in 2016. Traits with significant inbreeding depression are indicated with an asterisk (*).

There was no significant inbreeding depression in first year survival, or in whether plants flowered (survival: *X^2^ =* 0.01, *df* = 1, *P* > 90; proportion flowering: *X^2^* = 2.05, df = 1, *P* > 0.10; **Supplementary Table 1**; **Figures 5**, **6**). However, log-transformed time to flowering was 10% shorter in outcrossed than selfed families and inflorescence mass was 72% greater in outcrossed than selfed plants (flowering time; *X^2^* = 11.01, *df* = 1, *P* < 0.001; inflorescence mass; *X^2^* = 19.21, *df* = 1, *P* < 0.0001). We also detected a significant effect of competition environment on inflorescence mass in 2014 between plants with selfed and outcrossed competitors compared to plants with no competitor. Inflorescence mass in plants of both breeding treatments without a competitor was 40% greater than inflorescence mass in plants with a selfed competitor and 70% greater than the inflorescence mass of plans with an outcrossed competitor, respectively, with both statistically significant (*X^2^* = 10.86, *df* = 2, *P* < 0.01). No other response variables were affected by competition and there was no significant interaction effect of breeding by competition for inflorescence mass (**Supplementary Table 1**).

### Comparison of traits under field conditions

From 2015 through 2017, we periodically discarded focal plants that possessed selfed or outcrossed competitors if they became indistinguishable, owing to the production of multiple stems and intertwining between rhizomes and thus sample sizes decreased between years (*n* = 176, 174, 121 in 2015, 2016, 2017, respectively). Outcrossed plants in 2016 were 37% more likely to flower than selfed plants and outcrossed inflorescences in 2016 and 2017 were 82% and 97% heavier in mass than selfed inflorescences, respectively, regardless of whether they were in competitive treatments or not (proportion of plants flowering in 2016; *X^2^* = 8.21, *df* = 1, *P* < 0.01; inflorescence mass in 2016; *X^2^* = 5.00, *df* = 1, *P* < 05, inflorescence mass in 2017: *X^2^* = 7.05, *df* = 1, *P* < 0.01; **Supplementary Table 1**). Breeding treatment did not significantly affect other response variables. Plants of either breeding treatment with no competitor were 85% more likely to survive than plants with an outcrossed competitor in 2017 (*X^2^* = 8.2, *df* = 1, *P* < 0.05); however, survival of plants with selfed competitors was not significantly different from survival of plants with no competitors, or from plants with outcrossed competitors and there was no significant interaction between breeding and competitive treatment for survival (**Supplementary Table 1**).

### Analysis of growth via non-linear time-series

Growth data for *L. salicaria* grown under glasshouse conditions in 2014 best fit a Gompertz curve with an AIC of 10774.12 (**Supplementary Table 2**). The optimal nonlinear model parameters were: *Asymp* = 121.69 (se = 3.18), *b2 =* 4.28 (se = 0.07), *b3 =* 0.97 (se = 0.0009). We were able to successfully fit breeding treatment and competitive treatment to *Asymp* and *b2* in the 2014 model: the *Asymp* values in outcrossed plants were 18% greater than in selfed plants (*F* = 24.03, *df* = 1, *P* > 1.0x10^-5^) but *b2* did not differ significantly between breeding treatments (*F*-value = 0.00, *df* = 1, *P* > 0.95). Competitive environment did not significantly affect nonlinear growth parameters in 2014 (*Asymp*: *F* = 1.18, *df* = 2, *P* > 0.30; *b2*: *F* = 2.02, *df* = 2, *P* > 0.10). Inbreeding depression in *Asymp* was *δ* = 0.17, 95% CI: 0.11, 0.24).

In 2015-2016, plant growth most closely conformed to the Logistic model based on AIC (**Supplementary Table 2**). The model terms in 2015 were *Asymp* = 36.95 (se = 1.18), *xmid* = 59.79 (se = 0.61), *scal* = 12.57 (se = 0.88) and the 2016 terms were *Asymp* = 91.79 (se = 0.252), *xmid* =39.96 (se = 0.74), *scal* = 29.13 (se = 1.12). We were able to successfully test the effects of breeding treatment on the *Asymp* parameter of each model, but the models did not converge with treatments as covariates to other model terms. We found a significant effect of breeding treatment on the asymptote for 2016 with outcrossed plants having an *Asymp* 29% greater than selfed plants (*F* = 23.16, *df* = 1, *P* < 1.0x10^-5^); however, there was no difference in asymptotes between breeding treatments in 2015 (*F* = 3.36, *df* = 1, *P* > 0.05). There was a 23% higher Asymp term for plants with no competitor relative to those with an outcrossed competitor in 2016 (*F* = 4.9, *df* = 2, *P* < 0.01) but not in other competitive environments or in 2015 (*Asymp* in 2015: *F* = 1.46, *df* = 2, *P* > 0.20). We did not detect a significant effect of the interaction between breeding treatment and competitive environment on the *Asymp* value in 2016 (*Asymp* from the interaction of breeding treatment and competitive environment: *F* = 0.38, *df* = 2, *P* > 0.65). Inbreeding depression in *Asymp* from 2015 and 2016 was *δ* = 0.10, 95% CI: 0.0, 0.21, and *δ* = 0.23, 95% CI: 0.14, 0.31, respectively. These patterns indicate that growth curves differ in the field and glasshouse and possess different asymptotes.

**Figure 7:**
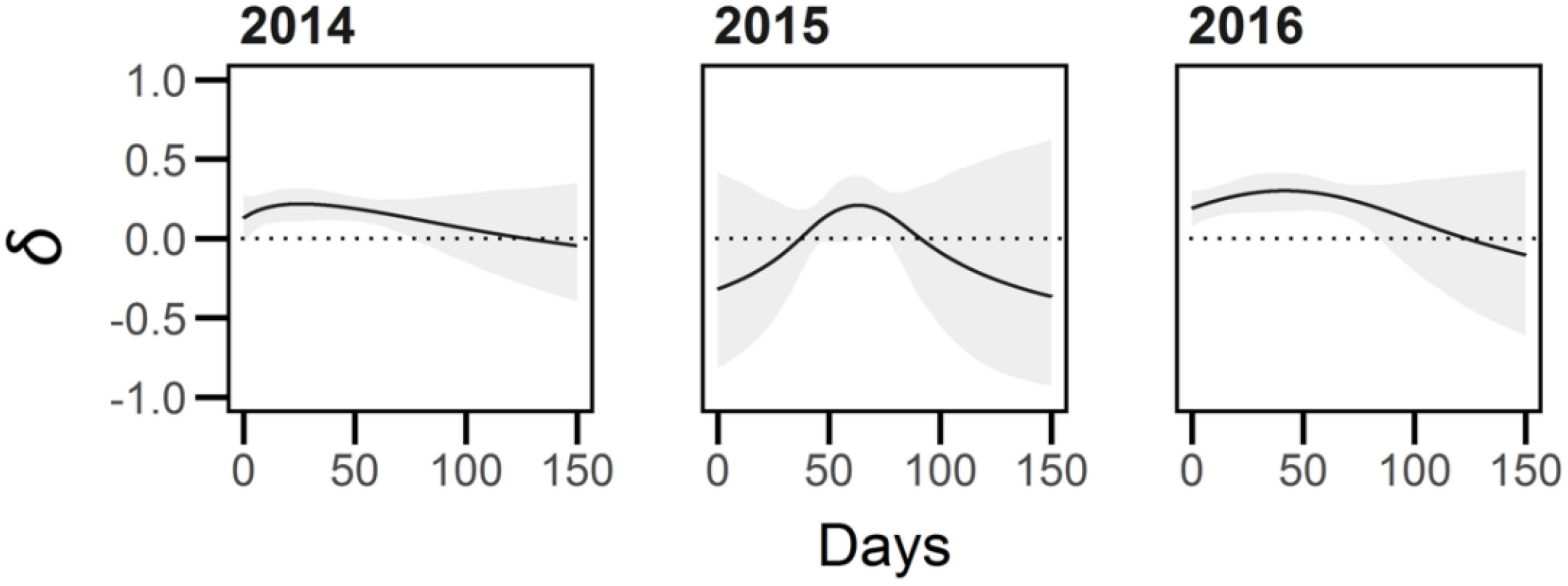
Inbreeding depression (*δ*) in average growth rate (*AGR*) experienced by *Lythrum salicaria* plants in the non-linear growth models. The grey shading represents the 95% confidence interval of estimates of *δ*. In 2014 and 2016 the outcrossed plants exhibited a significantly higher *AGR* relative to selfed plants early to mid-season (0.85 to 76.75 and 0 to 85.7 days, respectively), whereas in 2015 average growth rate was only significantly higher for a short time window mid-season (61.6 to 76.15 days). The difference in 2014 and 2016 caused a difference in asymptote (*Asymp*) whereas the difference in 2015 was not significant.

**Figure 8:**
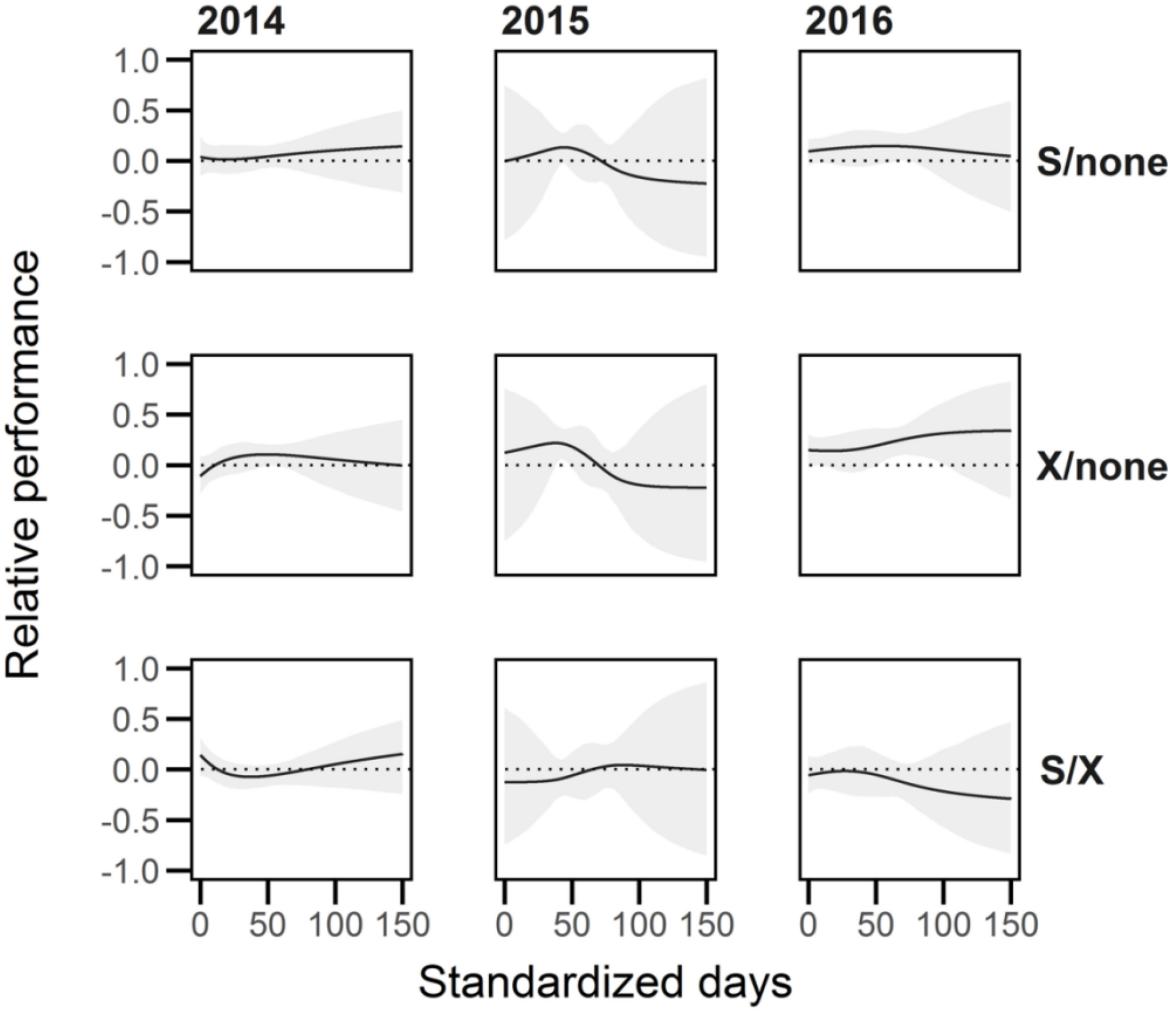
The relative performance (*RP*) of *Lythrum salicaria* plants represented as the average growth rate (*AGR*) between competitive environments (‘none’, selfed, outcrossed). We calculated the relative performance (where *AGR_1_* and *AGR_2_* represent the first and second competitive environment labelled to the right of each row in the plot) as *RP* = 1 - *AGR_1_*/*AGR_2_* when *AGR_2_* > *AGR_1_*, or *AGR_2_*/*AGR_1_* – 1 if *AGR_2_* < *AGR_1_*. There were some differences in average growth rate between outcrossed and no competitor for short periods in all years, and one short period in 2016 where the *AGR* of selfed over ‘none’ was significantly different from zero.

Inbreeding depression in average growth rate (*AGR*) during 2014 and 2016 was significantly above zero from day 0.85 to 76.75 and 0 to day 85.7, respectively, whereas in 2015 this ratio was only significantly different between day 61.6 and 76.15 (**Figure 7**). The ratios of competitive values differed between plants with an outcrossed competitor and plants with no competitor for only short periods of time in each year (between days 52.40-53.05 and 54.35-55.90 in 2014, 39.10-47.65 in 2015, and 52.90-99.20 in 2016), and only between the selfed competitor and no competitor treatments from days 57.90 – 75.50 in 2016 (**Figure 8**). There was no interaction between the breeding and competition treatments for inbreeding depression in AGR.

### Multiplicative function

**Figure 9:**
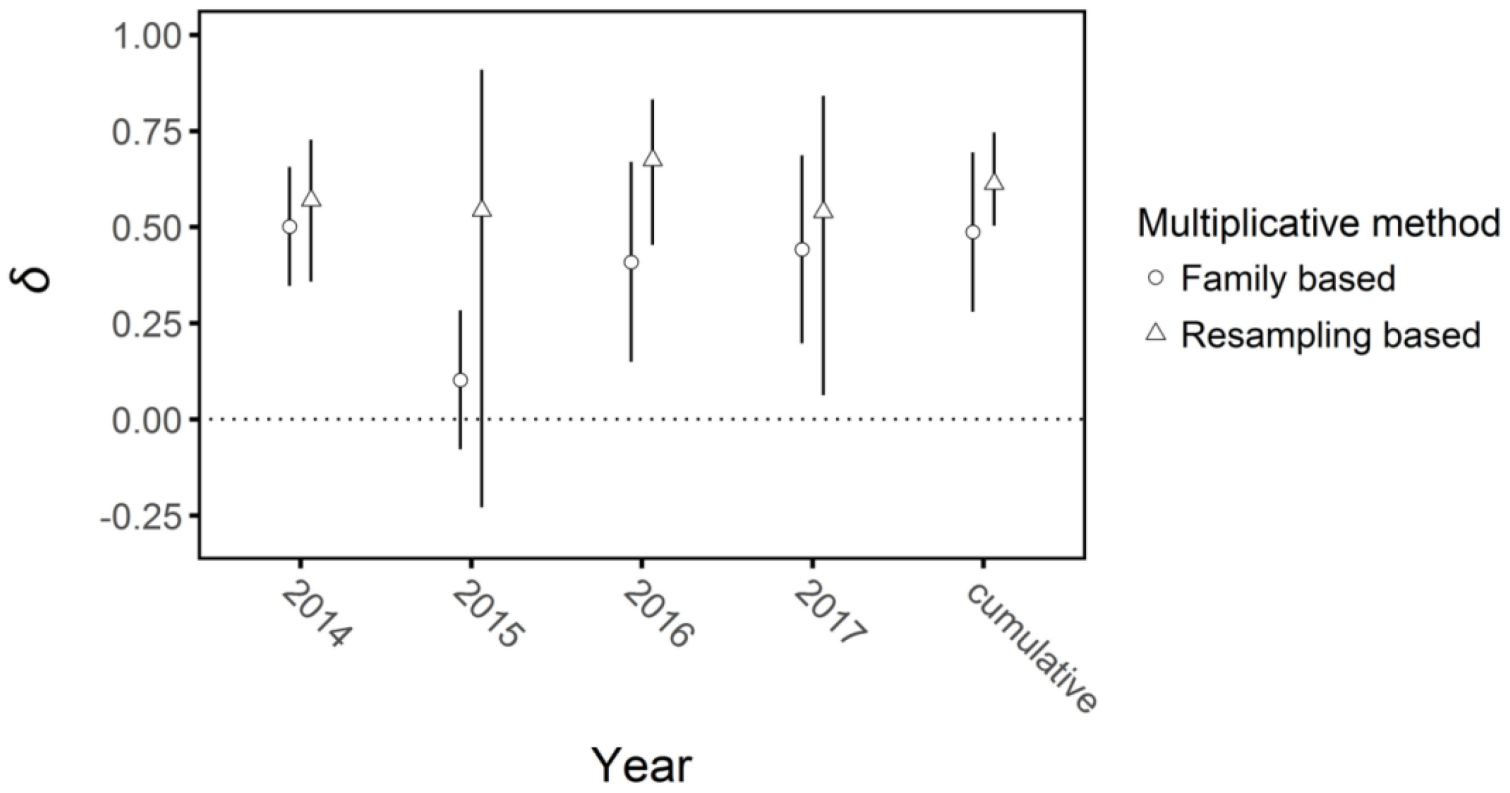
Multiplicative inbreeding depression (*δ*) in each year and cumulatively at the end of the four-year experiment on *Lythrum salicaria* with 95% confidence intervals (bars). The two values plotted were calculated based on family mean inbreeding depression and a resampling method from the observed distributions of data (see Methods). Overall, the two approaches gave similar values in each year and, with the exception of 2015, inbreeding depression was of similar magnitude among years.

The two metrics of multiplicative inbreeding depression were generally congruent (**Figure 9**). The family-based metric of mean inbreeding depression in 2014 when plants were grown under glasshouse conditions was *δ* = 0.50, 95% CI: 0.35, 0.66, which contrasted with the first year under field conditions (2015) when inbreeding depression did not differ significantly from zero (*δ* = -0.03, 95% CI: -0.31, 0.26). However, inbreeding depression became significant again in 2016 and 2017 (*δ* =0.40, 95% CI: 0.15, 0.67; *δ* = 0.44, 95% CI: 0.20, 0.69, respectively, **Figures 6**, **7**). Overall cumulative inbreeding depression from family-based estimates over all four years of the experiment was *δ* = 0.48, 95% CI: 0.28, 0.69.

Inbreeding depression via the resampling method gave similar results and additionally confirmed that the competition treatment did not strongly influence plant performance. The sampling based multiplicative inbreeding depression values for each year’s output matched generally, with the exception that the 2015 data had much greater variance (*δ* = 0.54, 95% CI: 0.23, 0.91). Cumulative inbreeding depression at the end of the experiment via resampling was slightly higher than the family-based values, though not significantly so (*δ* = 0.68, 95% CI: 0.51, 0.74). The values for multiplicative per-year and cumulative relative performance in all environments overlapped in all cases except for the cumulative value of plants with a selfed competitor versus those with no competitor (*RP* = 0.39, 95% CI: 0.03, 0.65, **Supplementary Figure 2**). These results indicate that there was no consistent overall effect of competitive treatment on inbreeding depression in the experiment.

## Discussion

In our experiment we detected a small amount of inbreeding depression in seed germination (*δ* = 0.12) and inconsistent inbreeding depression in survival and proportion of plants flowering. In contrast, at the end of three out of the four growing seasons reproductive output exhibited stronger inbreeding depression values of *δ* = 0.44, 0.45, and 0.49 in 2014, 2016 and 2017, respectively, and our analysis of relative growth rates indicated significant inbreeding depression in all three years. Overall, cumulative inbreeding depression based on several key life-history traits was *δ* = 0.48 or 0.68, depending on the method used. In contrast to our predictions, we found no consistent effects of the competitive environment (selfed or outcrossed competitor) on the magnitude of inbreeding depression. Below, we discuss the implications of these findings for the invasion biology of *L. salicaria* and whether inbreeding depression may affect the mating system and demography of colonizing populations. We also discuss some of the limitations of our experiment and how future work on inbreeding depression might be improved.

### Inbreeding depression during biological invasion

Biological invasions are punctuated by frequent founder events and population bottlenecks (Novak and Mack, 2005; Barrett et al., 2017), which can expose deleterious recessive alleles to selection and purging (Lande and Schemske, 1985; Kirkpatrick and Jarne, 2000) or result in their fixation resulting in reduced fitness (Keller and Waller, 2002). Despite the potential of these processes to induce a lag phase for an invasion or enable heterosis after secondary contact between invasive populations (Sakai et al., 2001; Roman and Darling, 2007; Keller et al., 2014), few studies have used experimental approaches to measure inbreeding depression or genetic load under field conditions in invasive populations.

Outcrossing populations are predicted to maintain a significant genetic load of deleterious recessive alleles, which when exposed through inbreeding should result in significant inbreeding depression (Lande and Schemske, 1985; Charlesworth and Willis, 2009). Our experiment confirmed this prediction as inbreeding depression was evident across a range of life-history traits and among the four years of the experiment. Based on the cumulative measures of inbreeding depression calculated over the entire experiment (**Figure 9**), selfed offspring of *L. salicaria* were on average roughly half as fit as outcrossed offspring, a pattern consistent with an earlier study on seedling growth traits (O’Neil, 1994), and consistent with the strength of selection predicted to oppose the evolution of selfing in the absence of selection for reproductive assurance (Fisher, 1941; Lloyd, 1979). Nevertheless, this level of inbreeding depression is significantly lower than has been reported in several other outcrossing perennial angiosperms (e.g. Kohn, 1988; Sakai et al., 1989; Eckert and Barrett, 1994; Lande et al., 1994; Vogler et al., 1999; Dorken et al., 2002). Future metanalysis of the strength of inbreeding depression in plant species with contrasting outcrossing rates, life-history traits and population histories would be valuable to identify the complex causes of variation in inbreeding depression.

One possible reason for the relatively moderate inbreeding depression in *L. salicaria* is that our experiment only lasted for four growing seasons and plants of the species may live for substantially longer. Studies of cumulative inbreeding depression over the entire life span of *L. salicaria* may reveal a higher intensity of inbreeding depression; inbreeding depression often intensifies through the life history and is more strongly expressed at later life stages (Husband and Schemske, 1996). However, it is worth noting there was no evidence that year-to-year measures of inbreeding depression intensified over the course of our experiment. To our knowledge, no studies have attempted to investigate cumulative inbreeding depression over the entire lifetime of long-lived perennials using experimental field approaches. This is presumably because of the logistical and resource demands that such a long-term experiment would entail. However, genetic marker-based estimates of inbreeding depression (see Ritland, 1990; Koelling et al., 2012) can be used in such a situation and these have been usefully applied to long-lived plant species (e.g. Eckert and Barrett, 1994; Dorken et al., 2002). Studies such as these provide a powerful way to examine inbreeding depression in the particular environment in which natural populations occur and avoid the problem of context dependency, a weakness of the more widely used experimental approach of Darwin (1876), which has been a shortcoming in many comparisons of fitness in selfed and outcrossed progeny conducted under glasshouse conditions.

A second possible reason that might explain why inbreeding depression was not especially severe in our experiment is because *L. salicaria* is a highly successful colonizing species and bottlenecks and periods of small population size are a pervasive feature of its population biology. These demographic events, as well as the possibility of bouts of biparental inbreeding in small populations, could serve to reduce genetic load and hence the intensity of inbreeding depression (Lande and Schemske, 1985; Kirkpatrick and Jarne, 2000; Pujol et al., 2009; Verhoeven et al., 2011; Peischl et al., 2013; Marchini et al., 2016). Comparisons of inbreeding depression between the native and invasive range of *L. salicaria*, between large and stable versus small and transient populations, or between populations with different invasion histories in the introduced range, should provide insight on the role of demographic factors in shaping the intensity of inbreeding depression in the species. In addition, future studies of inbreeding depression in a larger number of invasive populations of *L. salicaria* covering a broader geographical range would be valuable. Our geographically restricted and pooled sample of families from sites around Toronto ignored the possibility of population-specific local adaptation which would likely influence variation in the magnitude of inbreeding depression.

The third potential factor that might influence the intensity of inbreeding depression in *L. salicaria* is the autotetraploid nature of invasive populations in North America (Kubátová et al., 2008). There are theoretical arguments (Lande and Schemske, 1985; Bever and Felber, 1992) and empirical evidence (Husband and Schemske, 1997) that the strength of inbreeding depression in autotetraploids is likely to be less than in diploids because autotetraploid populations should experience slower progress to homozygosity than diploids per generation and therefore inbreeding will expose fewer recessive deleterious alleles (Lande and Schemske, 1985; Bever and Felber, 1992). Because both diploid and autotetraploid populations of *L. salicaria* occur in the native European range (Kubátová et al., 2008) it should be possible to evaluate the extent to which autopolyploidy might reduce the strength of inbreeding depression in *L. salicaria*. A study comparing diploid and tetraploid cytotypes of the invasive *Centaurea stoebe* found cumulative inbreeding depression in diploid but not tetraploid populations (Rosche et al., 2017). Significantly, diploids in this species are restricted to the native European range whereas tetraploids have become invasive in N. America, a similar pattern to *L. salicaria* implicating polyploidy in promoting colonizing success.

### Timing and cumulative effects of inbreeding depression

The timing of inbreeding depression during the life cycle is a key factor for understanding its overall expression and we addressed this topic with nonlinear growth curves. Nonlinear growth curves and estimates of relative growth rate have been used in models of plant performance, particularly involving the physiological responses of agricultural crops to genetic or environmental adversity (Chen et al., 2014), examination of the relations between metabolism versus size (Muller-Landau et al., 2006), the presence of trade-offs between growth rate and life history traits, including survival and reproduction after disturbance (Rose et al., 2009) or growth rate versus herbivore defences (Paul-Victor et al., 2010). However, to our knowledge this approach has not been used to study differences between the growth of selfed and outcrossed families in plant populations.

Our analyses revealed complex patterns that showed variation among years, with two showing consistent curves (2014, 2016) and another (2015) in which the timing and magnitude of inbreeding depression in average growth rate was quite different (**Figure 7**). It is not clear what mechanisms were responsible for this variation, especially since years 2015 and 2016 were both under field conditions. However, results in general for 2015, the first year of field conditions, differed from the remaining two years in showing only a relatively short widow of time in which outcrossed progeny outperformed selfed progeny. In 2014 and 2016 inbreeding depression was evident early to mid-season, which may be an indication that earlier-acting growth of outcrossed plants may be magnified and result in the large differences evident in end-of-season inflorescence mass. Future studies of inbreeding depression could usefully apply these methods to understand the timing of the effects of dominance and suppression between competing selfed and outcrossed progeny (Schmitt et al., 1987; Schmitt and Ehrhardt, 1990). Also, these approaches in conjunction with genetic mapping studies (see Charlesworth and Willis, 2009) could be used to quantify the time at which recessive deleterious alleles are expressed during plant growth potentially enabling the identification of loci governing inbreeding depression.

### Competition and inbreeding depression

Our experiment revealed no consistent effects of competition on the magnitude of inbreeding depression in *L. salicaria*. In contrast, several earlier plant studies have reported significant competitive effects on inbreeding depression (e.g. Schmitt et al., 1987; Cheptou et al., 2000), although this result is by no mean universal. Indeed, 16 of 20 studies on plants reported no overall pattern of increased inbreeding depression under intraspecific competitive stress (see Appendix 1b in Willi et al., 2007). Although there were a few sporadic significant differences in trait values between competitive environments (i.e. inflorescence mass in 2014, survival in 2017, cumulative multiplicative fitness; **Supplementary Table 1** and **Figure 2**), these differences usually only occurred between a subset of the competitive treatments and also lacked an interaction term between competition environment and breeding treatment. Other work of this type has exposed focal plants to a set of competitors (e.g. Schmitt et al., 1987; Schmitt and Ehrhardt, 1990; Wolfe, 1993; Cheptou and Schoen, 2003), whereas in our study we used a single competitor against the focal plant within a pot (**Figure 1**). Under the field conditions of this experiment this design may not have provided sufficient power for the detection of competitive differences between selfed and outcrossed plants.

One feature of our design may have been especially important in this respect. For practical considerations, we left all pots sunk into the ground for the entire duration of the field experiment (3 years). Therefore, plants in all pots were able to grow roots through the bottom of the pots into the surrounding soil and exploit available belowground resources. It is therefore likely that plants in the competitive treatments were not exposed to the full severity of competitive conditions that they would have faced in the more constrained below-ground environment of a single pot. Future research on the effects of competition on inbreeding depression in *L. salicaria* could usefully implement experimental designs in which a much higher density of competing plants is used than in the current study since invasive populations are often comprised of monospecific stands.

### Inbreeding depression and the maintenance of tristyly

Genes promoting self-fertilization can spread in outcrossing populations as a result of ‘automatic selection’ unless inbreeding depression is severe (Fisher, 1941; Lloyd, 1979; Lande and Schemske, 1985). The threshold value of inbreeding depression preventing the spread of selfing varies depending on a variety of genetic, demographic and reproductive factors (Lloyd, 1980; 1992; Uyenoyama et al., 1993; Goodwillie et al., 2005; Busch and Delph, 2012). But generally, if outcrossed offspring are more than twice as fit as selfed offspring, outcrossing in animal-pollinated species should be maintained as long as pollinator service is reliable. Although there is evidence that standing genetic variation in partial self-incompatibility is a common feature of *L. salicaria* populations (Balogh and Barrett, 2018b), there is no indication from the literature that any population of *L. salicaria* is fully self-compatible, or has transitioned to high selfing rates as a result of the breakdown of tristyly and evolution of semi-homostyly.

The absence of *L. salicaria* populations with high selfing rates is significant because this transition has occurred elsewhere in the genus *Lythrum* (Weller, 1992), and in other tristylous taxa as a result of the breakdown of tristyly to semi-homostyly (e.g. *Eichhornia* – Barrett, 1988; *Oxalis* – Ornduff, 1972). Although semi-homostyles have been described as sporadic variants in *L. salicaria* (Stout, 1925) they do not appear to form monomorphic selfing populations. Therefore, although demographic conditions associated with biological invasion might more generally favour the evolution of selfing from outcrossing in colonizing populations (Pannell, 2015), this transition has apparently not occurred in *L. salicaria*. This finding suggests that the maintenance of tristyly in small colonizing populations of *L. salicaria* populations occurs because any inbred offspring that do result from selfing are selected against owing to the general superiority of outcrossed offspring. Cumulative values of inbreeding depression over the four years of this study are generally consistent with this hypothesis.

## Data Availability

The original contributions presented in the study are included in the article/**Supplementary Material**. Further inquiries can be directed to the corresponding author.
