# Supplementary material for: An experimental field study of inbreeding depression in an outcrossing invasive plant
Source: Front Plant Sci. 2024 Aug 29;15:1393294. doi: 10.3389/fpls.2024.1393294 (PMC11390429; doi:10.3389/fpls.2024.1393294)
Supplement: Supplementary file 1 [file DataSheet1.pdf]

Supplementary Materials

Table 1 The likelihood-ratio significance values for harvest data collected from the glasshouse experiment on *Lythrum salicaria* in 2014 including germination, survival, flowering, flowering time and final inflorescence mass. All traits were tested by breeding treatment and competitive treatment and if a significant competitor effect for a trait was detected, I provide the significance of the interaction between breeding treatment and competitive environment.

Trait	Distribution	Treatment	Likelihood-ratio X^2	*df*	*P*-value	Time
Germination percent	Binomial	Breeding	10.99	1	< 0.001	early life
Survival before transplant	Binomial	Breeding	1.71	1	> 0.15	early life
Survival at harvest	Binomial	Breeding	0.01	1	> 0.90	2014
Flowering percent	Binomial	Breeding	2.05	1	> 0.15	2014

Flowering time	Continuous (log-transformed)	Breeding	11.01	1	< 0.001	2014
Mass of inflorescence	Continuous	Breeding	19.21	1	$< 1.2 \times 10^{-5}$	2014
Survival at harvest	Binomial	Breeding	0.51	1	> 0.47	2015
Flowering percent	Binomial	Breeding	3.17	1	> 0.05	2015
Flowering time	Continuous (log-transformed)	Breeding	0.23	1	> 0.63	2015
Mass of inflorescence	Continuous (log-transformed)	Breeding	1.34	1	> 0.24	2015
Survival at harvest	Binomial	Breeding	0.45	1	> 0.50	2016
Flowering percent	Binomial	Breeding	8.21	1	< 0.01	2016

Flowering time	Continuous (log-transformed)	Breeding	2.22	1	> 0.13	2016
Mass of inflorescence	Continuous (log-transformed)	Breeding	5	1	< 0.05	2016
Survival at harvest	Binomial	Breeding	3.33	1	> 0.05	2017
Flowering percent	Binomial	Breeding	3.64	1	> 0.05	2017
Flowering time	Continuous (log-transformed)	Breeding	1.49	1	> 0.22	2017
Mass of inflorescence	Continuous (log-transformed)	Breeding	7.05	1	< 0.05	2017
Survival at harvest	Binomial	Competition	2.99	2	> 0.22	2014
Flowering percent	Binomial	Competition	3.07	2	> 0.22	2014

Flowering time	Continuous (log-transformed)	Competition	0.67	2	> 0.72	2014
Mass of inflorescence	Continuous	Competition	10.86	2	< 0.01	2014
Survival at harvest	Binomial	Competition	0.83	2	> 0.65	2015
Flowering percent	Binomial	Competition	0.59	2	> 0.70	2015
Flowering time	Continuous (log-transformed)	Competition	1.37	2	> 0.50	2015
Mass of inflorescence	Continuous (log-transformed)	Competition	1.57	2	> 0.45	2015
Survival at harvest	Binomial	Competition	0.97	2	> 0.60	2016
Flowering percent	Binomial	Competition	1.55	2	> 0.45	2016

Flowering time	Continuous (log-transformed)	Competition	0.53	2	> 0.75	2016
Mass of inflorescence	Continuous (log-transformed)	Competition	1.96	2	> 0.35	2016
Survival at harvest	Binomial	Competition	8.2	2	< 0.05	2017
Flowering percent	Binomial	Competition	2.87	2	> 0.20	2017
Flowering time	Continuous (log-transformed)	Competition	3.05	2	> 0.22	2017
Mass of inflorescence	Continuous (log-transformed)	Competition	5.04	2	> 0.05	2017
Mass of inflorescence	Continuous	Breeding	6.67	1	< 0.01	2014
		Competition	5.3	2	> 0.05	

		Interaction	1.09	2	> 0.57	
		Breeding	1.75	1	> 0.18	
Survival at harvest	Binomial	Competition	2.99	2	> 0.22	2017
		Interaction	0.28	2	> 0.86	

Table 2 The AIC values for nonlinear model types to which each year of growth data for *Lythrum salicaria* was fit. The Gompertz model possesses the lowest AIC in 2014 whereas the Logistic model possessed the lowest AIC in 2015 and 2016.

Model	AIC values	Year
Four-part logistic	12128.9	2014
Gompertz	10774.12	2014
Logistic	11015.83	2014
monomolecular	12443.63	2014
Four-part logistic	Failed	2015
Gompertz	5200.62	2015
Logistic	5191.99	2015
Monomolecular	5346.67	2015

Four-part logistic	Failed	2016
Gompertz	5881.16	2016
Logistic	5872.04	2016
Monomolecular	5921.76	2016

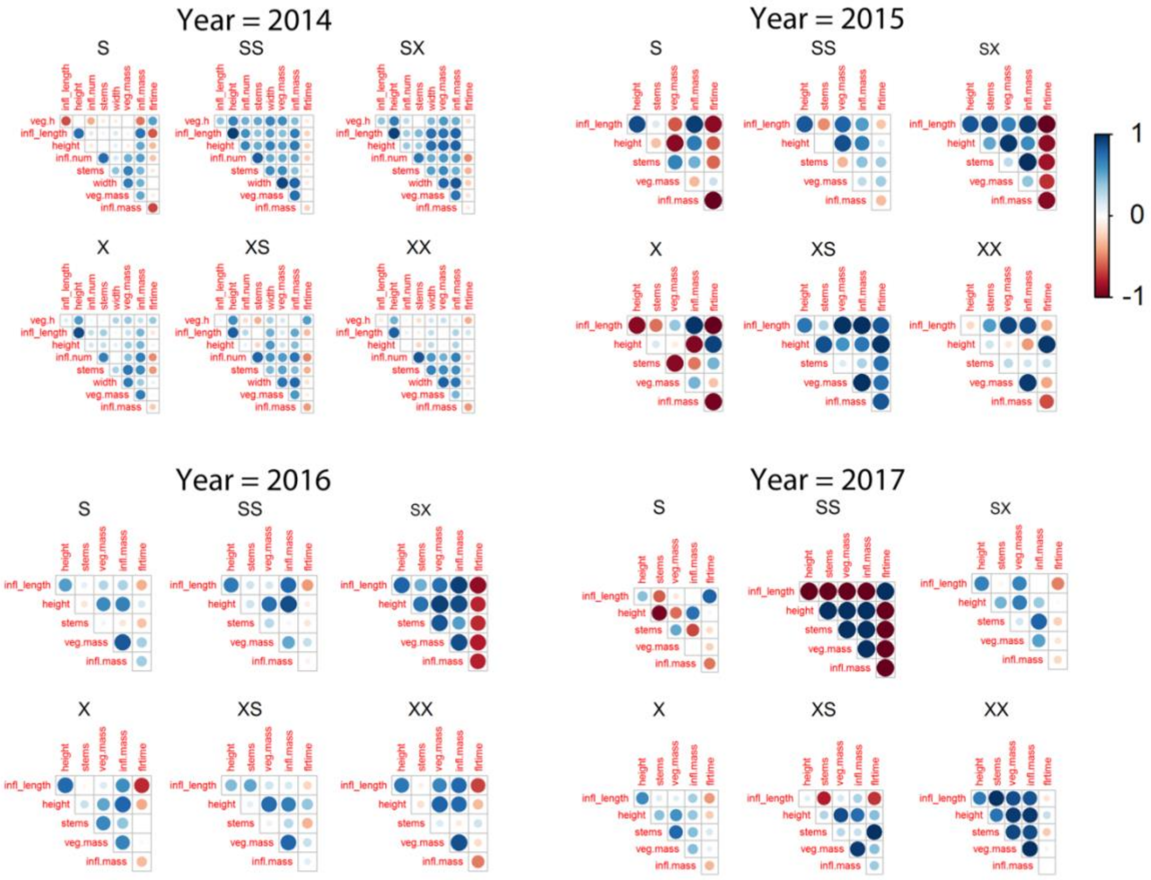

Figure 1 Correlation plots depicting the level of covariance between each measured trait in each of the six treatments and across each year observed in the inbreeding depression experiment on *Lythrum salicaria*. No correlations were consistently expressed between years and between treatments within years, which prohibited use of a single easily-measured trait or multivariate test in the analysis of inbreeding depression.

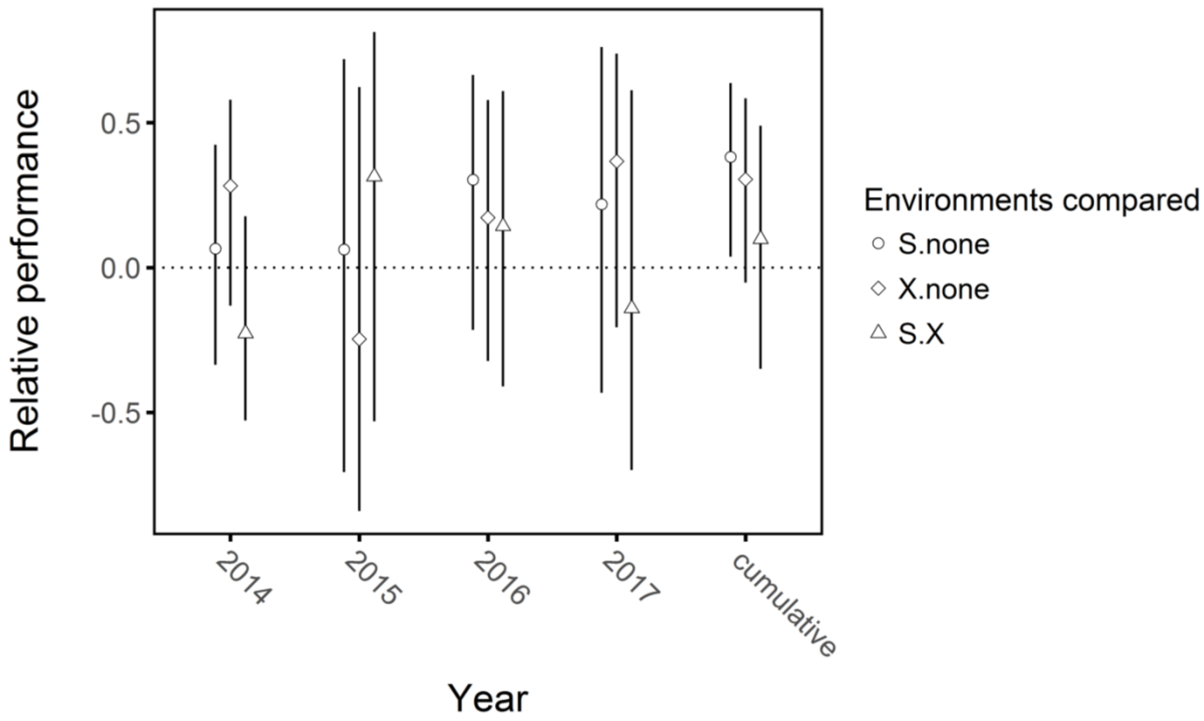

Figure 2 The multiplicative depiction of relative performance (*RP*) of plants of *Lythrum salicaria* in the different competitive environments of the study. The relative performance was calculated as $1 - ARG_1/AGR_2$ if $AGR_2 > AGR_1$ or $AGR_2/AGR_1 - 1$ if $AGR_2 < AGR_1$ with *AGR* equal to the mean multiplicative performance of plants with no competitor, a selfed competitor (S), or an outcrossed competitor (X). This measure could only be calculated from the resampling method due to unequal survival within families. In all but one case, the 95% confidence intervals of relative performance overlapped with zero. The exception occurred for cumulative performance of plants with a self-fertilized competitor, which performed slightly worse than those with no competitor.
